# Synthesis of 4-Methoxy-1, 3-Benzenediolylhydrazones and Evaluation of Their Anti-Platelet Aggregation Activity

**DOI:** 10.22037/ijpr.2019.1100856

**Published:** 2019

**Authors:** Chaoqing Wang, Yan Wang, Qingsong Deng, Xiujie Liu

**Affiliations:** a *School of Chemistry and Chemical Engineering, Tianjin University of Technology, Tianjin 300384, China. *; b *Tianjin Key Laboratory of Organic Solar Cells and Photochemical Conversion, School of Chemistry and *; c *Chemical Engineering Tianjin University of Technology, Tianjin 300384, China.*

**Keywords:** 4-Methoxy-1, 3-benzenediolylhydrazones, Anti-platelet aggregation, Cytotoxicity, Picotamide

## Abstract

In our present investigation, a series of novel 4-methoxy-1,3-benzenediolyl-hydrazones were designed and synthesized, and their ability to inhibit platelet aggregation was evaluated by adenosine diphosphate (ADP) and arachidonic acid (AA). The structures of the synthesized compounds were conﬁrmed by spectral data. Results demonstrated that the activities of all compounds excelled the positive drug Picotamide (25.1% inhibition rate) and seven compounds (**PNN01**,** PNN03**,** PNN05**,** PNN07**,** PNN09**,** PNN12**,** and PNN14**) have efficiently inhibited platelet aggregation even higher than Clopidogrel (37.6% inhibition rate) induced by AA. Among them, **PNN07 **(39.8% inhibition rate**) **was considered as the most potent analogue. Evaluation of cytotoxic activity of the compounds against L929 cell line revealed that none of the compounds have signiﬁcant cytotoxicity. Thus, diolylhydrazones derives are potential to be antiplatelet aggregation inhibitors and maybe working in AA-induced selectively.

## Introduction

Cardiovascular diseases (CVDs) account for over 17 million deaths globally each year (30% of all deaths) and the number is expected to grow to 23.6 million by 2030 (1). Most CVD patients are suffering from atherosclerosis, one due to many external factors, including physical insufficient physical activities, inadequate eating, and tobacco intake, and some internal risk factors, including diabetes, hypertension, and metabolic syndrome and so on (2). The rupture of atherosclerotic plaques and intracavitary thrombosis is the most common mechanism of most acute coronary syndromes and sudden coronary death, despite continued advances have been made in pharmacy and surgical treatments (3).

Platelet is a key participants in atherothrombosis (4, 5) due to their capacity to adhere to the injured blood vessel wall and amplify the procoagulant response (6, 7). In this process, a proaggregant signaling cascade is eventually triggered by plaque rupture, resulting in full platelet activation that leads to further aggregation and exposure of a procoagulant surface that enables the formation of a fibrin-rich hemostatic plug (8). In general, antithrombotic drugs, antiplatelet agents (eg. clopidogrel, aspirin®, tirofiban), are also the primary treatment options for these diseases. However, major limitation including inter-individual variability, increased risk of bleeding, neutropenia, thrombocytopenia, and drug resistance (9-13) need better and therapeutic drugs and in the same prompt the development of novel antiplatelet agents.

Picotamide (Figure 2 left), a novel antiplatelet agents, is a dual inhibitor of both TxA_2_ receptors and TxA_2_ synthase. In addition, *in-vitro* studies using rings of rat aorta demonstrated that Picotamide was unable to suppress PGI_2_, which is released from the endothelium and is generally inhibited by COX-1. This kind of dual action makes it possible to enhance therapeutic efficacy in the prevention of thrombosis, including inhibition of platelet aggregation and accumulation of antiaggregatory prostaglandins (PGI_2_ and PGD_2_) (14). Unfortunately, although it is safer than aspirin but still has a short half-life and weaker activity. However, it is these outstanding features of Picotamide that sparked our great interest so that over the past two decades, our research group has made many attempts to modify the structure of Picotamide based on the concept of bioisosterism. We envisioned replacing the two amide components in Picomaide with two ester components or two sulfonamide components and replacing the two 3-pycolyl groups in picotamide with two substituted phenyl groups in the same time. And we previously demonstrated a successful application of such analogues of Picomaide and showed that the derivatives exhibit excellent inhibitory effects *in-vitro* induced by ADP and AA (Figure 2 A, B, C) (15).

Several articles have been reported that N-acylheteroaryl hydrazones (NAH) had already become platelet aggregation inhibitors and other bioactive agents. Cunha *et al*., synthesized N-phenylpyrazolyl moiety which has the NAH chain exhibiting inhibitory effect on AA-, collagen, and ADP-induced platelet aggregation. So it represented a novel family of anti-platelet agents. Silva *et al.* in 2004 suggested that the hydrazone and acylhydrazone moieties demonstrate a subunit which stabilize free radicals that mimicking bis-allyl fragment of unsaturated fatty acids such as arachidonic acid (AA). Furthermore, these fragments have an important role as pharmacophore cores with anti-infammatory, anti-nociceptive, and anti-platelet aggregation activity (Figure 2 D) (16, 17).

Bezerra-Neto *et al*., (Figure 2E) (18) have previously described the N-acylhydrazone subunit as a pharmacophore group for analgesic, anti-inflammatory, and anti-platelet properties, and it is also considered as a privileged structure in the design of new bioactive compounds (19, 20). Coquelet *et al*. have reported some acylhydrazone derivatives as inhibitors of platelet cyclooxygenase (COX) and lipooxigenase (LOX) blocking the transformation of arachidonic acid to its proaggregatory metabolites. In light of the finding of this study, Barreiro *et al*., in a series of studies, managed to develop some new hydrazones which effectively inhibited platelet aggregation with selective inhibitory activity toward platelet aggregation induced by arachidonic acid (AA). They found active group of hydrazones with arylsulfonate acylhydrazone, phenothiazine-1-acylhydraz-one,

N-substituted-phenyl-1,2,3-triazole-4-acylhydrazone, and pyrazolylhydrazone structures. Although a diverse group of derivatives have been found to exhibit anti-platelet activity, in these studies they share a preserved structural backbone that is two (hetero) aromatic ring systems linked by a hydrazone bond (Figure 2F) (21).

Moreover, in a successful study, indole ring is demonstrated as a structural moiety which has anti-platelet aggregation effect. Indole-3carbinol, a natural compound found in cruciferous vegetables, is known to have anti-platelet and anti-thrombotic activities *in-vitro* and *in-vivo*. Indole-3-carbinol has been shown to inhibit collagen-induced platelet aggregation in human platelet-rich plasma (PRP) in dose-dependent manner (Figure 2G) (22).

**Scheme 1:**
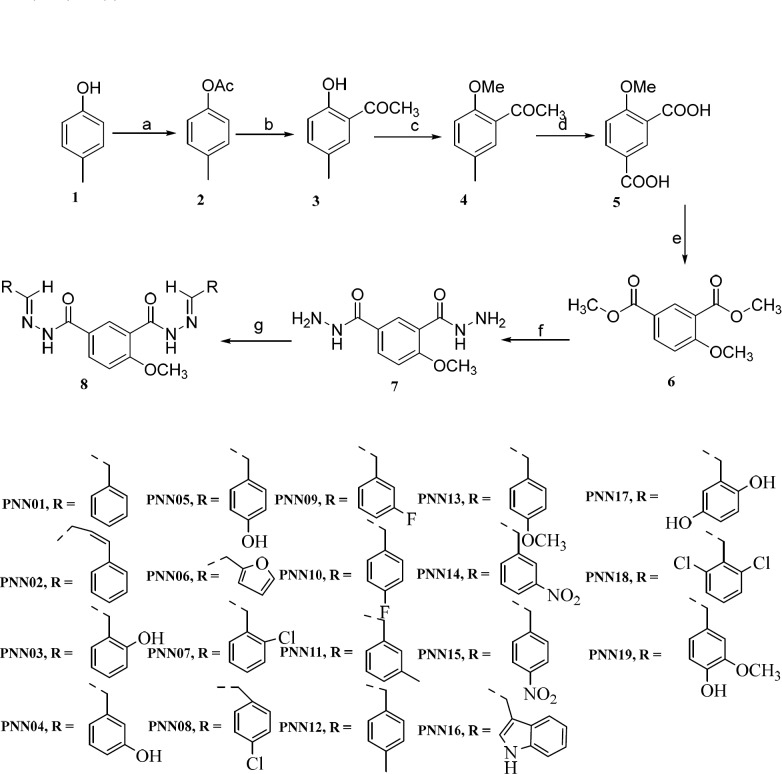
The concept of target compounds

Encouraged by these results of our previous studies on the biological properties of Picotamide′s derivatives and based on the above mentioned reports, in this investigation, nineteen derivatives of 4-methoxy-1,3- benzenediolyl hydrazones bearing two hydrazone moiety compounds as potential antiplatelet agents were rationally designed and synthesized (Scheme 1.).

## Custom

*Materials and instruments*

**Scheme 2:**
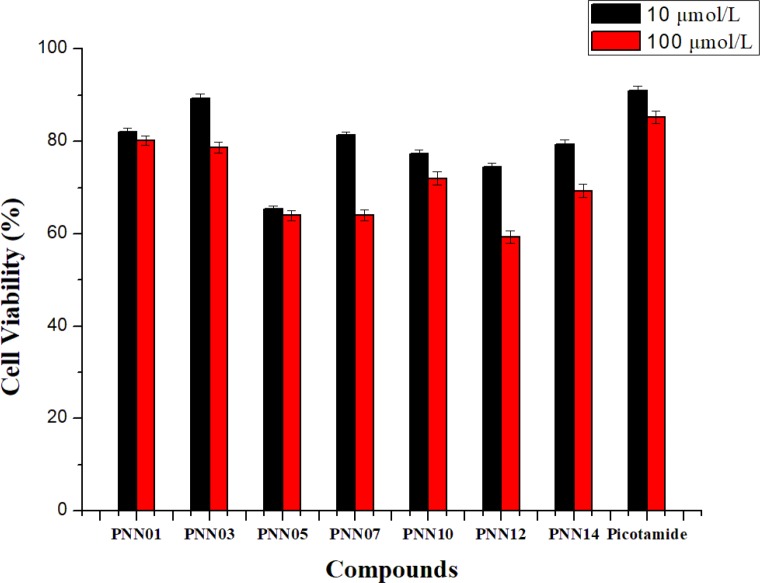
The synthesis pathway of target compounds: (a). Ac_2_O, refluxed 3 h (b). AlCl_3, _110 °C 8 h (c). NaOH, (MeO)_2_SO_2, _TBAB, 75 °C 4.5 h (d). KMnO_4, _NaOH, 80 °C (e). SOCl_2_, anhydrous methanol, 0 °C 1 h, 80 °C 7 h (f). 80% H_2_NNH_2_·H_2_O 80 °C 6 h (g). glacial acetic acid, aromatic aldehydes, refluxed 2-4 h

All chemical reagents were purchased from Aladdin Industrial Corporation (P.R. China), Energy Chemical (P.R. China), and Tianjin Hengshan (P.R. China) and used without further purification. The reagents of cell viability were purchased from Beyotime Biotechnology (P.R. China). Other biological reagents arachidonic acid (AA) was purchased from Sigma and also, adenosine diphosphate (ADP) was purchased from Solarbio life sciences. The melting points were determined with a Kofler micro melting point apparatus and were uncorrected. Nuclear magnetic resonance (^1^H NMR and ^13^C NMR) spectra were recorded on a Bruker 400 MHz spectrometers (Bruker, Rheinstetten, Germany), pick positions are illustrated in parts per million (d) in DMSO-d_6 _solution and TMS (0.05% v/v) as internal standard, and coupling constant values (J) are given in Hertz. Signal multiplicities are reported by: s (singlet), d (doublet), t (triplet), q (quadruplet), m (multiplet), and brs (broad signal). For NMR spectral data assignments, the atom numbering of compounds is depicted in Scheme 2. Analytical thin-layer chromatography (TLC) was performed with Merck silica gel plates and visualized with UV irradiation (254 nm). High-resolution mass spectra (HRMS) were recorded on an Agilent 6520B UPLC-Q-TOF mass spectrometer (Agilent Technologies, Santa Clara, CA, USA). Melting points were obtained by an Electrothermal 9100 apparatus and are uncorrected. The IR spectra were taken by a PerkinElmer 843 spectrometer with KBr as diluent.

*General procedure for the preparation of 4-methoxyisophthalic acid (5)*

Intermediates 2-5 were prepared according to the literature method (28).

Yield 69.76%, white solid, m.p. 260.3–261.7 °C; IR νcm^-1^: 3300.24 br (OH), 2955.31 (C-H), 1701.67 (C=O). ^1^H NMR (400 MHz, DMSO-d_6_) δ ppm: 3.87 (s, 3H, OCH_3_), 7.16 (d, 1H, Ar-H,* J* = 8.8 Hz), 8.03 (dd, 1H, Ar-H,* J *= 8.8 Hz, 2.0 Hz,), 8.23(d, 1H, Ar-H, *J* = 2.0 Hz), 12.90(s, 2H, COOH). ^13^C NMR (101 MHz, DMSO-*d*_6_) δ (ppm): 169.32, 169.25, 166.27, 136.14, 131.12, 129.43, 128.11, 127.33, 55.76; MS: m/z 195 [M –H]^–^.

*Dimethyl 4-methoxyisophthalate(6)*

Thionyl chloride (1.5 mL, 20.0 mmol) was added dropwise to a stirred solution of 4-methoxyisophthalic acid (5) (1.0 g, 5.0 mmol) in anhydrous methanol (50 mL) at 0 °C under nitrogen and reaction for 1 h. The reaction mixture was then allowed to stir at 80 °C for 7 h and monitored by TLC (PE: EA = 1:1, Rf = 0.67). Solvent was removed after completion of reaction to give a colorless crystalline solid, then dissolved in the solution of acetic ether (20 mL), and washed with cold 5 % NaHCO_3 _(aq) three or four times till the pH 7. Separating the oil phase, it was dried over anhydrous sodium sulfate (Na_2_SO_4_). After filtering the desiccant, the solution was concentrated under rotary evaporator to obtain isophthalic dimethyl ester as yield as white solid 1.0 g, yield: 87.7%; m.p. 134.1-135.5; ^1^H NMR (400 MHz, DMSO-*d*_6_) δ (ppm): 8.24 (d, *J* = 2.3 Hz, 1H, H-2), 8.11 (dd, *J* = 8.8, 2.3 Hz, 1H, H-6), 7.29 (d, *J* = 8.8 Hz, 1H, H-5), 3.91 (s, 3H, Ar-OCH_3_), 3.82 (d, *J* = 10.6 Hz, 6H, 2×OCH_3_); ^13^C NMR (101 MHz, CDCl_3, _TMS) δ 165.94, 165.66, 162.44, 134.95, 133.42, 122.13, 119.96, 113.60, 56.20, 52.05; IR (cm^-1^) : 3129.18(υ_CH_), 1712.25 (υ_C=0_); HR ESI-MS: [M+H]^+ ^m/z % 224.0690, 

Calcd for C_11_ H_12_ O_5_ , 224.0688.

*Methoxyisophthalohydrazide (7)*

To a solution of dimethyl 4-methoxyisophthalate (6) (0.5 g, 2.23 mmol) in 5 mL of anhydrous ethanol and 80% hydrazine monohydrate (4.19 g, 40.94 mL 83.71 mmol) was added. The reaction mixture was kept in 80 ℃ for 6 h, and the progress of the reaction was monitored by TLC. After completion of the reaction, the solvent was concentrated and the mixture was cooled. The precipitate obtained was filtered off, and recrystallized from water affording the desired compound **7** white needle crystal 0.43g, yield 86 %; m.p. > 300 ℃; ^1^H NMR (400 MHz, DMSO-d_6_) δ (ppm): 9.74 (s, 1H, C=ONH), 9.28 (s, 1H, C=ONH), 8.14 (d, *J* = 2.2 Hz, 1H, H-2), 7.92 (dd, *J* = 8.7, 2.3 Hz, 1H, H-6), 7.16 (d, *J* = 8.7 Hz, 1H, H-5), 4.53 (brs, 4H, 2×NH_2_), 3.89 (s, 3H, OCH_3_); ^13^C NMR (101 MHz, DMSO-d_6_) δ (ppm): 168.32, 165.85, 161.81, 132.94, 127.05, 124.62, 119.37, 118.62, 56.58; IR (cm^-1^) : 3415.53 (υ_NH2_), 3298.79 (υ_NH_), 1636.92 (υ_C=0_), 1498.83 (υ_C=N_); HR ESI-MS: [M+H]^+ ^m/z % 224.0910, Calcd for C_9_H_12_N_4_O_3_, 224.0908.

*The general procedures for preparation of target compounds* ***8*** *, taking* ***PNN01*** *as an example*

A solution of hydrazide compound **7 **(0.5g, 2.20 mmol) in 10 mL glacial acetic acid was added to 3.0 amount of appropriate benzaldehyde (0.71g, 0.68 mL, 6.70 mmol) slowly. Reaction mixture was heated under reflux with stirring for about 3 h monitored by TLC (DCM : MeOH = 15 : 1, Rf = 0.61) and poured into ice/water mixture. The precipitate was filtered and washed with cold water at first then ethanol to give white solid, recrystallized from DMF-H_2_O to obtain beige solide 0.42g, yield 67.42%, m.p. = 223.1-224.8 (Scheme 2). The other compounds were prepared in the similar manner.

*N′* ^1^ *,N′* ^3^ *-di((E)-benzylidene)-4-methoxyisophthalohydrazide (PNN01 )*

Yield 67.42%, beige solide, m.p. 223.1-224.8 ℃; ^1^H NMR (400 MHz, DMSO-*d*_6_) δ (ppm): 11.90 (s, 1H, C=ONH), 11.63 (s, 1H, C=ONH), 8.47 (d, *J* = 10.8 Hz, 1H, N=CH), 8.36 (s, 1H, N=CH), 8.23 (s, 1H, H-2), 8.12 (d, *J* = 2.2 Hz, 1H, H-6), 7.74 (d, *J* = 6.0 Hz, 5H, Ar-H), 7.47 (d, *J* = 6.7 Hz, 5H, Ar-H), 7.33 (s, 1H, J = 8.7 Hz, H-5), 3.96 (s, 3H, OCH_3_). ^13^C NMR (101 MHz, DMSO-*d*_6_)δ(ppm): 169.61, 162.38, 159.83, 146.81, 135.02, 134.60, 133.72, 132.53, 129.93, 129.28, 128.55, 125.62, 123.90, 118.25, 117.42, 56.77; IR (cm^-1^) : 3133.88 (υ_NH_), 1639.38 (υ_C=O_), 1618.25 (υ_C=N_), 1270.42 (υ_N-N=C_); HR ESI-MS: [M+H]^+ ^m/z % 401.1538, Calcd for C_23_H_20_N_4_O_3_, 401.1536.

*4-methoxy-N′1-((1E,2E)-3-phenylallylidene)-N′3-((E)-3-phenylallylidene)isophthalohydrazide (PNN02)*

Yield 62.13%; white flocculent crystals, m.p. 249.2-251.9 ℃; ^1^H NMR (400 MHz, DMSO-*d*_6_) δ (ppm): 11.80 (s, 1H, C=ONH), 11.51 (s, 1H, C=ONH), 8.27 (d, *J* = 6.6 Hz, 1H, H-6), 8.21 (s, 1H, H-2), 8.12 (dd, *J* = 14.7, 8.1 Hz, 2H, 2×N=CH), 7.88 (d, *J* = 10.1 Hz, 1H, CH=CH), 7.61 (s, 4H, Ar-H), 7.54 (d, *J* = 7.0 Hz, 1H, CH=CH), 7.38 (d, *J* = 6.9 Hz, 3H, H-5), 7.32 (s, 3H, Ar-H), 7.27 (d, 1H,* J* = 6.7 Hz, H-5), 7.06 (s, 2H, CH=CH), 3.95 (s, 3H, OCH_3_); ^13^C NMR (101 MHz, DMSO-*d*_6_) δ (ppm): 162.12, 159.71, 150.05, 139.64, 139.31, 136.36, 132.42, 129.75,129.20, 127.50, 126.08, 125.58, 123.95, 117.39, 56.72; IR (cm^-1^) : 3133.55 (υ_NH_), 1638.64 (υ_C=O_), 1537.74 (υ_C=N_), 1268.22 (υ_N-N=C_); HR ESI-MS: [M+H]^+ ^m/z % 452.1852, Calcd for C_27_H_24_N_4_O_3_, 452.1848.

*N′1-((E)-2-hydroxybenzylidene)-N′3-(2-hydroxybenzylidene)-4-methoxyisophthalohydrazide (PNN03)*

Yield 65.22%; yellow crystals, m.p. 268.4-270.1 ℃; ^1^H NMR (400 MHz, DMSO-*d*_6_) δ 12.19 (s, 1H, C=ONH), 11.86 (s, 1H, C=ONH), 11.37 (s, 1H, OH), 11.28 (s, 1H, OH), 8.68 (s, 1H, N=CH), 8.60 (s, 1H, N=CH), 8.33 (d, 1H, J = 8.6 Hz, H-6), 8.15 (s, 1H, H-2), 7.54 (d, *J* = 5.6 Hz, 2H, Ar-H), 7.31 (s, 3H, H-5, Ar-H), 6.95 (s, 4H, Ar-H), 3.97 (s, 3H, OCH_3_). ^13^C NMR (101 MHz, DMSO-*d*_6_) δ (ppm): 162.77, 162.08, 160.03, 157.98, 157.04, 148.85, 147.87, 144.76, 132.79, 132.38, 130.07, 129.28, 127.96, 125.82, 125.17, 123.39, 119.83, 119.12, 117.01, 56.80. IR (cm^-1^): 3415.23 (υ_OH_), 3130.28 (υ_NH_), 1617.77 (υ_C=O_), 1544.28, 1487.49 (υ_C=N_), 1280.13 (υ_N-N=C_); HR ESI-MS: [M+H]^+ ^m/z % 432.1437, Calcd for C_23_H_20_N_4_O_5_, 432.1434.

*N′1-((E)-3-hydroxybenzylidene)-N′3-(3-hydroxybenzylidene)-4-methoxyisophthalohydrazide (PNN04)*

Yield 64.31%; gray crystals, m.p. 257.3-259.1 ℃; ^1^H NMR (400 MHz, DMSO-*d*_6_) δ (ppm): 11.82 (s, 1H, C=ONH), 11.55 (s, 1H, C=ONH), 9.64 (s, 2H, 2 × OH), 8.38 (d, *J* = 8.7 Hz, 1H, H-6), 8.25 (s, 1H, N=CH), 8.21 (s, 1H, N=CH), 8.10 (d, *J* = 6.9 Hz, 1H, H-2), 7.31 (d, *J* = 8.8 Hz, 1H, H-5), 7.26 (t, *J* = 7.8 Hz, 3H, Ar-H), 7.20 (s, 2H, Ar-H), 7.10 (d, *J* = 7.6 Hz, 2H, Ar-H), 6.83 (s, 1H, Ar-H), 3.96 (s, 3H, OCH_3_); ^13^C NMR (101 MHz, DMSO-*d*_6_) δ (ppm): 162.76, 162.10, 160.23, 157.99, 157.21, 148.84, 144.82, 132.79, 132.36, 130.11, 129.30, 127.89, 125.76, 125.21, 123.38, 119.80, 119.15, 117.32, 56.82; IR (cm^-1^): 3413.74 (υ_OH_), 3142.99 (υ_NH_), 1638.35 (υ_C=O_), 1555.89, 1493.14 (υ_C=N_), 1274.84 (υ_N-N=C_); HR ESI-MS: [M+H]^+ ^m/z % 432.1438, Calcd for C_23_H_20_N_4_O_5_, 432.1434.

*N′1-((E)-4-hydroxybenzylidene)-N′3-(4-hydroxybenzylidene)-4-methoxyisophthalohydrazide (PNN05* ***)***

Yield 70.13%; beige powder, m.p. 257.3-259.1 ℃; ^1^H NMR (400 MHz, DMSO-*d*_6_) δ (ppm): 11.74 (s, 1H, C=ONH), 11.44 (s, 1H, C=ONH), 9.97 (s, 2H, 2 × OH), 8.37 (d, *J* = 7.7 Hz, 1H, H-6), 8.24 (d, *J* = 10.5 Hz, 2H, N=CH), 8.09 (d, *J* = 8.5 Hz, 1H, H-2), 7.57 (d, *J* = 8.1 Hz, 4H), 7.31 (d, *J* = 8.7 Hz, 1H), 6.85 (d, *J* = 7.9 Hz, 4H, Ar-H), 3.94 (s, 3H, OCH_3_); ^13^C NMR (101 MHz, DMSO-*d*_6_) δ (ppm): 162.20, 160.17, 159.91, 159.67, 159.55, 148.60, 132.24, 129.81, 129.43, 128.74, 125.82, 124.14, 116.27, 56.76; IR (cm^-1^): 3411.31 (υ_OH_), 3134.46 (υ_NH_), 1643.23 (υ_C=O_), 1553.41 (υ_C=N_), 1271.75 (υ_N-N=C_); HR ESI-MS: [M+H]^+ ^m/z % 432.1435, Calcd for C_23_H_20_N_4_O_5_, 432.1434.

*N′1-((E)-furan-2-ylmethylene)-N′3-(furan-2-ylmethylene)-4-methoxyisophthalohydrazide (PNN06)*

Yield 73.42%; dark brown powder, m.p. 271.9-272.5 ℃; ^1^H NMR (101 MHz, DMSO-*d*_6_) δ (ppm): 11.85 (s, 1H, C=ONH), 11.57 (s, 1H, C=ONH), 8.35 (s, 1H, N=CH), 8.24 (s, 1H, H-2), 8.18 (s, 1H, N=CH), 8.09 (d, *J* = 7.5 Hz, 1H, H-6), 7.86 (d, *J* = 5.2 Hz, 2H, Furan-H), 7.31 (d, *J* = 8.6 Hz, 1H, H-5), 6.93 (d, *J* = 2.9 Hz, 2H, Furan-H), 6.64 (s, 2H, Furan-H), 3.94 (s, 3H, OCH_3_); ^13^C NMR (101 MHz, DMSO-*d*_6_) δ (ppm): 162.75, 162.18, 159.75, 149.89, 145.65, 137.96, 129.72, 125.52, 124.20, 118.3, 114.12, 113.82, 112.92, 112.34, 56.75; IR (cm^-1^): 3144.98 (υ_NH_), 1641.19 (υ_C=O_), 1566.06 (υ_C=C_), 1541.20 (υ_C=N_), 1276.17 (υ_N-N=C_); HR ESI-MS: [M+H]^+ ^m/z % 380.1128, Calcd for C_19_H_16_N_4_O_5_, 380.1126.

*N’1-((E)-2-chlorobenzylidene)-N’3-(2-chlorobenzylidene)-4-methoxyisophthalohydrazide (PNN07)*

Yield 80.67%; white powder, m.p. 292.8-293.4 ℃; ^1^H NMR (101 MHz, DMSO-*d*_6_) δ (ppm): 12.12 (s, 1H, C=ONH), 11.90 (s, 1H, C=ONH), 8.88 (s, 1H, N=CH), 8.75 (s, 1H, N=CH), 8.24 (s, 1H, H-2), 8.15 (d, *J* = 8.5 Hz, 1H, H-6), 8.07 – 8.00 (m, 1H, Ar-H), 7.52 (s, 2H, Ar-H), 7.45 (dd, *J* = 8.0, 4.4 Hz, 5H, Ar-H), 7.33 (d, *J* = 8.7 Hz, 1H, H-5), 3.96 (s, 3H, OCH_3_); ^13^C NMR (101 MHz, DMSO-*d*_6_) δ (ppm): 162.58, 162.36, 159.83, 144.02, 141.05, 134.7, 133.63, 131.98, 130.36, 128.04, 127.38, 126.62, 125.85, 118.32, 117.82, 56.76; IR (cm^-1^): 3137.61 (υ_NH_), 1650.02 (υ_C=O_), 1501.92 (υ_C=N_), 1263.45 (υ_N-N=C_), 758.49 (υ_C-Cl_); HR ESI-MS: [M+H]^+ ^m/z % 468.0762, Calcd for C_23_H_18_C_l2_N_4_O_3_, 

468.0760.

*N′1-((E)-4-chlorobenzylidene)-N′3-(4-chlorobenzylidene)-4-methoxyisophthalohydrazide (PNN08)*

Yield 80.67%; white powder, m.p. 292.8-293.4 ℃; ^1^H NMR (101 MHz, DMSO-*d*_6_) δ (ppm): 12.02 (s, 1H, C=ONH), 11.75 (s, 1H, C=ONH), 8.47 (s, 1H, N=CH), 8.34 (s, 1H, N=CH), 8.21 (s, 1H, H-2), 8.12 (d, *J* = 8.4 Hz, 1H, H-6), 7.54 (d, *J* = 8.3 Hz, 4H, Ar-H), 7.31 (d, *J* = 9.5, 7.2 Hz, 4H, Ar-H), 7.22 – 7.11 (m, 1H, H-5), 3.96 (s, 3H, OCH_3_); ^13^C NMR (101 MHz, DMSO-*d*_6_) δ (ppm): 162.25, 162.20, 159.69, 148.20, 140.34, 140.21, 132.39, 131.90, 130.00, 129.55, 127.52, 126.91, 125.78, 124.08, 118.36, 117.85, 56.72; IR (cm^-1^): 3135.56 (υ_NH_), 1641.90 (υ_C=O_), 1552.13 (υ_C=N_), 1270.14 (υ_N-N=C_), 814.51 (υ_C-Cl_); HR ESI-MS: [M+H]^+ ^m/z % 468.0761, Calcd for C_23_H_18_C_l2_N_4_O_3_, 468.0760.

*N’1-((E)-3-fluorobenzylidene)-N’3-(3-fluorobenzylidene)-4-methoxyisophthalohydrazide (PNN09)*

Yield 82.57%; gray flocculent crystals, m.p. 251.5-252.2 ℃; ^1^H NMR (101 MHz, DMSO-*d*_6_) δ (ppm): 11.95 (s, 1H, C=ONH), 11.67 (s, 1H, C = ONH), 8.46 (s, 1H, N=CH), 8.34 (s, 1H, N = CH), 8.21 (s, 1H, H-2), 8.11 (d, *J* = 8.5 Hz, 1H, H-6), 7.76 (d, *J* = 8.5 Hz, 4H, Ar-H), 7.53 (d, *J* = 8.4 Hz, 4H, Ar-H), 7.32 (d, *J* = 8.8 Hz, 1H, H-5), 3.96 (s, 3H, OCH_3_); ^13^C NMR (101 MHz, DMSO-*d*_6_) δ (ppm): 162.78, 162.52, 162.10, 159.85, 148.26, 135.39, 131.90, 130.00, 129.55, 127.52, 126.91, 125.78, 124.08, 118.32, 117.81, 114.35, 114.26, 56.72; IR (cm^-1^): 3157.74 (υ_NH_), 1650.88 (υ_C=O_), 1579.27 (υ_C=N_), 1265.94 (υ_N-N=C_); HR ESI-MS: [M+H]^+ ^m/z % 436.1352, Calcd for C_23_H_18_F_2_N_4_O_3_, 436.1350.

*N′1-((E)-4-fluorobenzylidene)-N′3-(4-fluorobenzylidene)-4-methoxyisophthalohydrazide (PNN10)*

Yield 85.66%; gray crystals, m.p. 250.2-251.1 ℃; ^1^H NMR ^1^H NMR (101 MHz, DMSO-*d*_6_) δ (ppm): 11.93 (s, 1H, C=ONH), 11.65 (s, 1H, C=ONH), 8.47 (s, 1H, N=CH), 8.35 (s, 1H, N=CH), 8.22 (s, 1H, H-2), 8.11 (d, *J* = 8.5 Hz, 1H, H-6), 7.82 – 7.76 (m, 4H, Ar-H), 7.31 (t, *J* = 8.8 Hz, 4H, Ar-H), 7.18 (d, *J* = 8.7 Hz, 1H, H-2), 3.96 (s, 3H, OCH_3_); ^13^C NMR ^1^H NMR (101 MHz, DMSO-*d*_6_) δ (ppm): 164.85, 164.79, 162.39, 162.32, 159.76, 147.04, 131.80, 131.48, 131.45, 131.34, 131.31, 129.79, 125.66, 124.03, 117.82, 112.43, 56.77; IR (cm^-1^): 3163.47 (υ_NH_), 1644.35 (υ_C=O_), 1556.02 (υ_C=N_), 1283.43 (υ_N-N=C_); HR ESI-MS: [M+H]^+ ^m/z % 436.1354, Calcd for C_23_H_18_F_2_N_4_O_3_, 436.1350.

*4-methoxy-N′1-((E)-3-methylbenzylidene)-N′3-(3-methylbenzylidene)isophthalohydrazide (PNN11)*

Yield 85.66%; white powder, m.p. 271.4-272.0 ℃; ^1^H NMR (101 MHz, DMSO-*d*_6_) δ (ppm): 11.88 (s, 1H, C=ONH), 11.63 (s, 1H, C=ONH), 8.43 (s, 1H, N=CH), 8.29 (s, 1H, N=CH), 8.20 (s, 1H, H-2), 8.11 (d, *J* = 8.4 Hz, 1H, H-6), 7.57 (s, 2H, Ar-H), 7.51 (d, *J* = 7.5 Hz, 2H, Ar-H), 7.34 (dd, *J* = 17.8, 8.4 Hz, 4H, Ar-H), 7.27 (d, *J* = 8.6Hz, 1H), 3.95 (s, 3H, OCH_3_), 2.37 (s, 6H, 2 × CH_3_); ^13^C NMR (101 MHz, DMSO-*d*_6_) δ (ppm): 162.34, 162.31, 159.74, 148.16, 138.56, 138.54, 134.77, 134.74, 132.38, 131.27, 129.78, 129.20, 127.80, 125.05, 117.65, 56.75, 21.36, 21.32; IR (cm^-1^): 3104.52 (υ_NH_), 1619.93 (υ_C=O_), 1589.50 (υ_C=N_), 1273.56 (υ_N-N=C_); HR ESI-MS: [M+H]^+ ^m/z % 428.1853, Calcd for C_25_H_24_N_4_O_3_, 428.1852..

*4-methoxy-N′1-((E)-4-methylbenzylidene)-N′3-(4-methylbenzylidene) isophthalohydrazide (PNN12)*

Yield 82.34%; white crystal, m.p. 249.1-250.4 ℃; ^1^H NMR (101 MHz, DMSO-*d*_6_) δ (ppm): 11.84 (s, 1H, C=ONH), 11.58 (s, 1H, C=ONH), 8.43 (s, 1H, N=CH), 8.30 (s, 1H, N=CH), 8.21 (d, *J* = 2.3Hz, 1H, H-2), 8.10 (d, *J* = 8.6 Hz, 1H, H-6), 7.63 (d, *J* = 7.9 Hz, 4H, Ar-H), 7.31 (d, *J* = 7.8 Hz, 1H, H-5), 7.28 (d, *J* = 7.6 Hz, 4H, Ar-H), 3.95 (s, 3H, OCH_3_), 2.35 (s, 6H, 2 × CH_3_); ^13^C NMR (101 MHz, DMSO-*d*_6_) δ (ppm): 162.22, 162.19, 159.68, 148.16, 148.06, 140.44, 132.30, 131.86, 129.93, 129.91, 129.74, 127.68, 127.61, 126.72, 126.94, 125.72, 117.67, 56.75, 21.47, 21.42; IR (cm^-1^): 3133.33 (υ_NH_), 1669.40 (υ_C=O_), 1536.74 (υ_C=N_), 1269.06 (υ_N-N=C_); HR ESI-MS: [M+H]^+ ^m/z % 428.1854, Calcd for C_25_H_24_N_4_O_3_, 428.1852.

***4-*** *Methoxy-N′1-((E)-4-methoxybenzylidene)-N′3-(4-methoxybenzylidene) isophthalohydrazide (PNN13)*

Yield 82.34%; white powder, m.p. 221.6-223.4 ℃; ^1^H NMR (101 MHz, DMSO-*d*_6_) δ (ppm): 11.76 (s, 1H, C=ONH), 11.49 (s, 1H, C=ONH), 8.41 (s, 1H, N=CH), 8.28 (s, 1H, N=CH), 8.19 (s, 1H, H-2), 8.09 (d, *J* = 8.4 Hz, 1H, H-6), 7.67 (d, *J* = 8.5 Hz, 4H, Ar-H), 7.31 (d, *J* = 8.6 Hz, 1H, H-5), 7.03 (d, *J* = 8.5 Hz, 4H, Ar-H), 3.95 (s, 3H, OCH_3_), 3.81 (s, 6H, 2 × Ar-0CH_3_); ^13^C NMR (101 MHz, DMSO-*d*_6_) δ (ppm): 162.16, 162.09, 161.36, 161.07, 159.63, 148.05, 132.19, 129.71, 129.12, 127.33, 125.85, 118.35, 117.74, 114.80, 114.78, 56.76, 55.73; IR (cm^-1^): 3127.76 (υ_NH_), 1683.84 (υ_C=O_), 1503.34 (υ_C=N_), 1282.00 (υ_N-N=C_); HR ESI-MS: [M+H]^+ ^m/z % 460.1753, Calcd for C_25_H_24_N_4_O_5_, 460.1751.

*4-methoxy-N′1-((E)-3-nitrobenzylidene)-N′3-(3-nitrobenzylidene) isophthalohydrazide (PNN14)*

Yield 87.87%; white powder, m.p. 288.1-288.5 ℃; ^1^H NMR (101 MHz, DMSO-*d*_6_) δ (ppm): 12.18 (s, 1H, C=ONH), 11.90 (s, 1H, C=ONH), 8.58 (s, 1H, H-2), 8.56 (s, 2H, N=CH), 8.47 (d, *J *= 8.7Hz, 1H, H-6), 8.28 (d, *J* = 7.5 Hz, 2H, Ar-H), 8.16 (t, *J* = 7.6 Hz, 4H, Ar-H), 7.77 (t, *J* = 7.9 Hz, 2H, Ar-H), 7.35 (d, *J* = 8.8 Hz, 1H, H-5), 3.97 (s, 3H, OCH_3_); ^13^C NMR (101 MHz, DMSO-*d*_6_) δ (ppm): 162.40, 162.32, 159.67, 148.48, 145.60, 136.47, 136.30, 133.61, 133.57, 131.74, 130.24, 130.11, 126.61, 126.15, 125.74, 121.29, 118.27, 117.76, 56.61; IR (cm^-1^): 3137.94 (υ_NH_), 1639.99 (υ_C=O_), 1527.29 (υ_C=N_), 1273.22 (υ_N-N=C_); HR ESI-MS: [M+H]^+ ^m/z % 490.1241, Calcd for C_23_H_18_N_6_O_7_, 490.1239.

**Figure 1:**
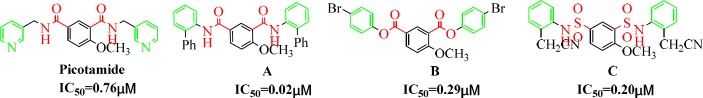
Our previous works: A. One of the most potent compounds of 4-methoxy-1,3-benzenedicarboxamides series. B. The most potent compound of 4-methoxy diphenyl isophthalates series. C. One of the most potent compounds of 4-methoxy-1,3- benzenedisulfonamides series

**Figure 2:**
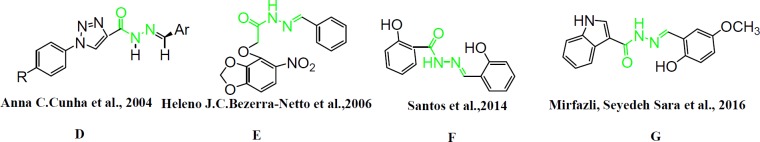
Reported antiplatelet compounds with hydrazone moiety pharmacophores

**Figure 3:**
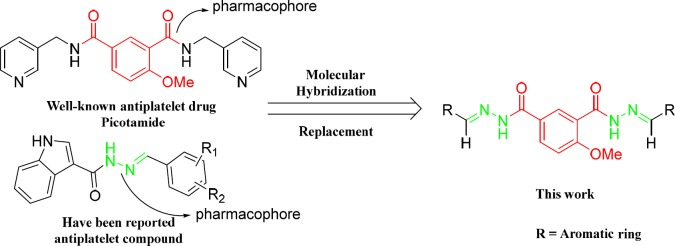
Cell viability of selected compounds and picotamide against fibroblast L929 cell line after 24 h of exposure

**Table 1:** Antiplatelet aggregation activities of nineteen derivatives of 4-methoxy-1,3-benzenediolylhydrazones

**Groups**	**n**	**Dose (μmol/L)**	**Inhibition (%)**	
			**AA**	**ADP**
Control (DMSO)	3	-	**-**	**-**
PNN01	3	1.3	38.7±1.2*,†,**	8.8±2.5*
PNN02	3	1.3	34.1±2.4*,**	10.1±1.8*
PNN03	3	1.3	38.2±1.8*,†,**	15.6±1.2*
PNN04	3	1.3	37.2±3.3*,**	-3.3±2.3
PNN05	3	1.3	38.5±1.7*,†,**	8.6±2.8*
PNN06	3	1.3	37.2±2.6*,**	11.8±3.1*
PNN07	3	1.3	39.8±2.1*,†,**	15.8±2.7*
PNN08	3	1.3	30.1±3.4*,**	-3.1±1.9
PNN09	3	1.3	36.1±1.9*,**	-12±2.0
PNN10	3	1.3	38.3±3.2*,†,**	-6.1±3.2
PNN11	3	1.3	28.2±2.9*,**	-5.3±3.6
PNN12	3	1.3	37.6±3.0*,†,**	-10.0±2.2
PNN13	3	1.3	28.0±3.4*,**	27.0±1.8*
PNN14	3	1.3	37.8±2.6*,†,**	25.0±2.1*
PNN15	3	1.3	30.6±1.8*,**	0.4±1.9
PNN16	3	1.3	27.1±2.7*,**	0.4±2.2
PNN17	3	1.3	26.0±1.4*,**	-0.8±2.3
PNN18	3	1.3	32.5±1.8*,**	7.9±2.1*
PNN19	3	1.3	29.3±2.3*,**	8.6±1.9*
Clopidogrel	3	1.3	37.6±3.2	55.1±2.8
Picotamide	3	1.3	25.1±2.5	40.4±2.0

Clopidogrel and Picotamide as positive drugs.

Data are expressed as the means±standard errors of the means. Statistical differences between the experimental and control groups were evaluated by analysis of variance followed by the Tukey test. * *p *< 0.05 vs. the control group; **† ***p *< 0.01 vs. Picotamide. ** *p *< 0.05 vs. Picotamide; ADP, adenosine diphosphate; AA, arachidonic acid.

*4-methoxy-N′1-((E)-4-nitrobenzylidene)-N′3-(4-nitrobenzylidene) isophthalohydrazide (PNN15)*

Yield 89.16%; yellow powder, m.p. 295.5-296.0 ℃; ^1^H NMR (101 MHz, DMSO-*d*_6_) δ (ppm): 12.18 (s, 1H, C=ONH), 11.88 (s, 1H, C=ONH), 8.50 (s, 1H, N=CH), 8.41 (s, 1H, N=CH), 8.36 (s, 1H, H-2), 8.27 (dd, *J* = 15.8, 7.6 Hz, 4H, Ar-H), 8.15 (d,* J* = 8.5 Hz, 1H, H-6), 7.97 (d, *J* = 8.4 Hz, 4H, Ar-H), 7.33 (d, *J* = 8.7 Hz, 1H, H-5), 3.98 (s, 3H, OCH_3_); ^13^C NMR (101 MHz, DMSO-*d*_6_) δ (ppm): 162.32, 162.30, 159.77, 148.05, 147.98, 145.49, 145.41, 140.82, 140.76, 130.85, 126.56, 126.11, 125.16, 125.03, 124.17, 123.96, 123.26, 118.26, 117.74, 56.56; IR (cm^-1^): 3135.25 (υ_NH_), 1677.48 (υ_C=O_), 1511.86 (υ_C=N_), 1261.85 (υ_N-N=C_); HR ESI-MS: [M+H]^+ ^m/z % 490.1243, Calcd for C_23_H_18_N_6_O_7_, 490.1239. 

***2*** *(N′3-((1H-indol-3-yl)methylene)-N′1-((E)-(1H-indol-3-yl)methylene)-4-methoxyisophthalohydrazide) (PNN16)*

Yield 80.79%; brown powder, m.p. 275.5-277.2 ℃; ^1^H NMR (101 MHz, DMSO-*d*_6_) δ (ppm): 11.60 (s, 2H, 2 × Indol-NH), 11.53 (s, 1H, C=ONH), 11.33 (s, 1H, C=ONH), 8.67 (s, 1H, N=CH), 8.53 (s, 1H, N=CH), 8.33 (s, 2H, Ar-H), 8.27 (s, 1H, H-2), 8.12 (d, *J* = 8.2 Hz, 1H, H-6), 7.82 (d, *J* = 6.5 Hz, 2H, Ar-H), 7.46 (d, *J* = 7.5 Hz, 1H, H-5), 7.35 – 7.28 (m, 2H, Ar-H), 7.19 (dd, *J* = 15.3, 8.1 Hz, 4H, Ar-H), 3.96 (s, 3H, OCH_3_); ^13^C NMR (101 MHz, DMSO-*d*_6_) δ (ppm): 161.55, 161.46, 159.10, 144.93, 144.72, 137.22, 137.13, 131.46, 130.49, 130.34, 126.72, 126.03, 125.63, 122.82, 122.17, 120.55, 120.15, 118.27, 117.76, 111.86, 111.75, 56.35; IR (cm^-1^): 3174.93 (υ_NH_), 1603.86 (υ_C=O_), 1574.91 (υ_C=N_), 1277.80 (υ_N-N=C_); HR ESI-MS: [M+H]^+ ^m/z % 478.1759, Calcd for C_27_H_22_N_6_O_3_, 478.1756. 

*N′1-((E)-2,5-dihydroxybenzylidene)-N′3-(2,5-dihydroxybenzylidene)-4-methoxyisophthalohydrazide (PNN17)*

Yield 75.86%; dark brown powder, m.p. 285.3-287.0 ℃; ^1^H NMR (101 MHz, DMSO-*d*_6_) δ (ppm): 12.04 (s, 1H, C=ONH), 11.73 (s, 1H, C=ONH), 10.36 (s, 2H, 2 × Ar-2-OH), 9.00 (s, 2H, 2 × Ar-5-OH), 8.58 (s, 1H, N=CH), 8.47 (s, 1H, N=CH), 8.25 (s, 1H, H-2), 8.12 (d, *J* = 9.0 Hz, 1H, H-6), 7.33 (d, *J* = 8.9 Hz, 1H, H-5), 6.97 (d, *J* = 5.7 Hz, 3H, Ar-H), 6.74 (d, *J* = 4.8 Hz, 3H, Ar-H), 3.96 (s, 3H, OCH_3_); ^13^C NMR (101 MHz, DMSO-*d*_6_) δ (ppm): 162.11, 161.86, 159.75, 150.57, 150.12, 148.60, 148.14, 147.99, 132.49, 129.76, 125.08, 123.32, 119.38, 119.18, 118.91, 117.42, 117.38, 117.16, 56.58; IR (cm^-1^): 3411.62 (υ_OH_), 3137.83 (υ_NH_), 1641.94 (υ_C=O_), 1540.17 (υ_C=N_), 1275.96 (υ_N-N=C_); HR ESI-MS: [M+H]^+ ^m/z % 464.1342, Calcd for C_23_H_20_N_4_O_7_, 464.1338. 

*N′1-((E)-2,6-dichlorobenzylidene)-N′3-(2,6-dichlorobenzylidene)-4-methoxyisophthalohydrazide (PNN18)*

Yield 89.64%; white powder, m.p. 314.2-315.3℃; ^1^H NMR (101 MHz, DMSO-*d*_6_) δ (ppm): 12.17 (s, 1H, C=ONH), 12.09 (s, 1H, C=ONH), 8.67 (s, 1H, N=CH), 8.55 (s, 1H, N=CH), 8.26 (d, *J* = 8.6 Hz, 1H, H-6), 8.08 (m, 2H, Ar-H and H-2), 7.58 (s, 3H, Ar-H), 7.45 (d, *J* = 7.9 Hz, 3H, Ar-H), 7.35 (d, *J* = 7.5 Hz, 1H, H-5); ^13^C NMR (101 MHz, DMSO-*d*_6_) δ (ppm): 162.24, 162.21, 159.77, 134.18, 134.12, 133.89, 133.84, 131.33, 131.21, 131.01, 130.68, 130.04, 129.12, 128.10, 127.23, 118.26, 117.70, 56.57; IR (cm^-1^): 3182.87 (υ_NH_), 1654.64 (υ_C=O_), 1556.31 (υ_C=N_), 1267.82 (υ_N-N=C_); HR ESI-MS: [M+H]^+ ^m/z % 535.9985, Calcd for C_23_H_16_Cl_4_N_4_O_3_, 535.9983. 

*N′1-((E)-4-hydroxy-3-methoxybenzylidene)-N′3-(4-hydroxy-3-methoxybenzylidene)-4-methoxyisophthalohydrazide (PNN19)*

Yield 70.08%; white powder, m.p. 216.2-217.3 ℃; ^1^H NMR (101 MHz, DMSO-*d*_6_) δ (ppm): 11.72 (s, 1H, C=ONH), 11.46 (s, 1H, C=ONH), 9.57 (s, 2H, 2 × Ar-OH), 8.36 (s, 1H, N=CH), 8.23 (s, 1H, N=CH), 8.08 (s, 1H, H-6), 7.92 (s, 1H, H-2), 7.41-7.20 (m, 6H, Ar-H), 6.85 (d, *J* = 8.6 Hz, 1H, H-5), 3.93 (s, 3H, OCH_3_), 3.83 (s, 6H, 2 × Ar-OCH_3_); ^13^C NMR (101 MHz, DMSO-*d*_6_) δ (ppm): 161.97, 161.86, 159.38, 149.29, 149.22, 148.64, 148.46, 148.29, 131.88, 130.87, 126.02, 125.86, 125.67, 125.24, 122.36, 118.28, 117.72, 112.02, 56.46, 55.81; IR (cm^-1^): 3134.16 (υ_NH_), 1637.42 (υ_C=O_), 1515.32 (υ_C=N_), 1271.16 (υ_N-N=C_); HR ESI-MS: [M+H]^+ ^m/z % 492.1652, Calcd for C_25_H_24_N_4_O_7_, 492.1647.

## Results and Discussion

*Chemistry*

The synthetic procedures to prepare these desired 4-methoxy-1,3-benzenediolyl hydrazones are shown in Scheme 2 4-Methylphenol **1 **was used as starting material. Acetylation of **1** with acetic anhydride gave 4-methylphenyl acetate **2** which was subjected to Fries rearrangement to 5-methyl-2-hydroxyacetophenone **3** in the presence of AlCl_3_. The crude product yield was near 90% over two steps. Compound **3** was reacted with dimethyl sulfate in aqueous sodium hydroxide using tetrabutylammonium bromide (TBAB) as catalyst to produce 2-methoxy-5-methylacetophen-one **4** in 78.5% yield. Oxidation of the methyl and acetyl groups in compound 4 afforded 4-methoxyisophthalic acid **5** which can be isolated in the pure state by recrystallization from ethanol. Dimethyl-4- methoxyisophthalate **6** was obtained by reacting **5 **in the presence of methanol with thionyl chloride as catalyst. The key intermediates 4-methoxyisophthalohydrazide **7 **were obtained by hydrazinolysis of **6** and in 85.2% yield, using hydrazine monohydrate 80% in ethanol. The final 4-Methoxy-1,3-benzenediolylhydrazone derivatives **9** were collected by condensing the hydrazide intermediates with the proper aromatic aldehydes in glacial acetic acid as the solvent refluxed for several minutes, in good yields. Synthesis of Schiff bases was performed in glacial acetic acid. This reaction in many cases was straightforward, but the intermediates **7** and products were all dissoluble in ethanol even in refluxed so their separation was difficult. Thus, glacial acetic acid was used as solvent and heated to complete the reaction.

*Biological Evaluation*

*In-vitro evalution of anti-platelet aggregation activity*

The *in-vitro* antiplatelet aggregation activities of nineteen derivatives of 4-methoxy-1,3- benzenedicarboxamides were measured by the turbidimetric method of Born and Cross (23). Blood was collected from anesthetized rats by venous puncture using syringes into tubes containing 3.8% sodium citrate (1:9, v/v). Platelet aggregation was assessed in platelet-rich plasma (PRP), obtained by centrifugation of citrated whole blood at room temperature for 10 min (500-800 rpm). The aggregation rate was measured by platelet aggregation analyzer after stimulation with AA (5 μM) and ADP using platelet-poor plasma (PPP) set to zero. The PPP was obtained by centrifugation of PRP at room temperature for 15 min (3000 rpm). A solution of the compounds (1.3 μmol/L) in DMSO (5 μL) was added into PRP (200 μL), and the same volume of DMSO without test compound was added to a reference sample (according to a pre-experiment, 5 μL of DMSO appears to have no significant effect on the platelet aggregation). After incubating for 2 min, the platelet aggregation was assessed and the percentage inhibition of platelet aggregation was calculated and the aggregation was monitored for 5 min. DMSO (0.5% v/v) was used as negative control and Picotamide and Clopidogrel as positive drugs. 

The results were expressed as mean±SD of 3 independent experiments. The platelet aggregation inhibition (%) was calculated by the following formula:

Inhibition % = (1-D/S) × 100%;

where D = platelet aggregation in the presence of test compounds, and S = platelet aggregation in the presence of solvent. The primary screening data for all compounds (1.3 μM) *in-vitro* activities on antiplatelet aggregation of the synthesized compounds are given in Table 1**. **The Statistical analysis was performed with ANOVA followed by Tukey′s test.

*Cytotoxicity effect on L-929 cells (* Figure 3 *)*

Mouse fibroblast cells (L929) were chosen to evaluate the *in-vitro* cytotoxicity of the materials and the drugs via Cell Counting Kit-8 (CCK-8) assays. L929 was cultivated in a humidified 5% carbon dioxide atmosphere at 37 °C on 96-well microplates, with 1×10^4 ^cells per well immersed in complete growth medium. The cells with 100 mL of RPMI-1640 per well were allowed to be attached for 24 h. Subsequently, the cells were then exposed to target compounds at a range of concentrations at 37 °C for 48 h. Target compounds concentration of 10 and 100 μmol L^-1 ^were added to L929 cells. After incubation for 48 h, the medium was removed and replaced with 100 μL of fresh complete medium of RPMI-1640. Then, CCK-8 solution was added to the 96-well plates at 10 μL per well and incubated for a further 30 min, and the absorbance at 450 nm was measured on a microplate reader (Bio-Tek FLx800 fluorescence microplate reader). This process was repeated eight times in parallel. The results are expressed as the relative cell viability (%) with respect to control wells, calculated as follows (24)**.**

Cell viability (%) = Abs (test cell) / Abs (controlled cell) × 100%

## Discussion

*Chemsitry*

The synthetic pathway is disclosed in Scheme 2. Final desired N-acylhydrazones were prepared through a classic imine formation reaction between 4-methoxy- isophthalohydrazide and different aromatic aldehydes. The ^1^H NMR and HR ESI-MS data of compounds approved the exact structures. In the ^1^H NMR spectra of these compounds, the existence of two singlets at 11.0 – 12.0 ppm was assigned to hydrazide C = ONH. Singlet signal at 8.2 – 8.9 ppm was assigned to N=CH. Molecular mass of all the derivatives was detected by high-resolution electron spray ionization mass spectrometry (HR ESI–MS) as M+1 relating to hydrogen of the intact molecules.

The acylhydrazone structures gave two sets of signals in NMR spectra whose intensities depend on solvent and the pair of signals coalesces on warming the NMR tube. The existence of the carbonyl oxygen atom and the imine nitrogen atom in N-acylhydrazones indicates geometrical isomers (E/Z). Syakaev *et al*. also noticed that derivatives of acylhydrazones from aromatic aldehydes showed two rotamers (cis and trans) due to the N–C (O) bond (25, 26).

In our study, the ^1^H NMR spectra at room temperature for some compounds of target compounds represented E/Z isomerization which was related to CH = N double bonds. Right after dissolution of the synthesized compounds in DMSO-*d*_6_, the double signals of E_C=N_/Z_C=N _conformers are registered by NMR. The two conformers show two sets of signals at different chemical shifts, and integration of the two sets of signals indicates that the E_C=N _conformer is the predominant conformer. Based on the earlier report that N-acylhydrazones derived from aromatic aldehydes in solution remained mostly in the E form because of the hindered rotation on the imine bond (27), we thought that E geometry in our cases.

*Anti-platelet aggregation activity*

The *in-vitro *antiplatelet activities of all synthesized compounds were assayed on rat platelet rich plasma (PRP) by using the Born′s turbidimetric method. ADP and Arachidonic acid (AA) were employed as inducers of platelet aggregation; both Picotamide and Clopidogrel were used as the positive controls. Inhibition rate values were calculated for the ability of the test compounds. The antiplatelet aggregation activity of the derivatives is listed in Table 1. Data show that the majority of the derivatives inhibited AA-induced platelet aggregation more effectively than the aggregation induced by ADP and also, some compounds showed inhibitory effects compared to clopidogrel. Among the tested compounds, derivatives **PNN01, PNN03, PNN05, PNN07, PNN09, PNN12 **and** PNN14 **showed high inhibition values and **PNN07 **was the most potent compound with inhibition value of 39.8% induced by AA. Among all the target compounds, on their substituted benzene rings, based on the activity data, it could be suggested that the most active compounds were among the electron-donating group structures. Hence, electronic properties of the aromatic ring may possibly contribute to the activity of the compounds and with one substitute is more potent than two. Just like the effects of electronic factors, there is no obvious rule for the size of substitutes on the anti-platelet aggregation activity.

*Cytotoxicity effect on L-929 cells*

The calculated cytotoxicity effect on L-929 cells of seven compounds **PNN01**,** PNN03**, **PNN05**,** PNN07**, **PNN09**,** PNN12 **and** PNN14** are given in Figure 3. The data analysis demonstrated that at lower concentration of 10 μmol/L, three compounds **PNN01**, **PNN03,** and **PNN07** have lower effect on L-929 cells, and the effect of **PNN03** is close to the control drug Picotamide. At the concentration of 100 μmol/L, the effects of three compounds are better than those of Picotamide. 

## Conclusion

We have rationally designed and synthesized nineteen analogues of Picotamide **PNN01-PNN19**. It was found that all the diolylhydrazones derivatives are having platelet aggregation inhibitory effect. In addition, comparing the activity of compounds against platelet aggregation induced by AA shows that compounds **PNN01, PNN03, PNN05, PNN07, PNN09, PNN12, and PNN14 **have significant antiplatelet aggregation and even higher than Clopidogrel (37.6%). However, when induced by ADP all the compounds showed little activities and lower than Picotamide (40.4%). So, the tested derivatives selectively inhibited platelet aggregation induced by AA with very good inhibition rates values. Among them, compound **PNN07** with inhibition value of 39.8% proved to be the most potent derivative of this series. Evaluation of cytotoxic activity of the compounds against L929 cell line revealed that none of the compounds have signiﬁcant cytotoxicity. Thus, we confirmed that diacylhydrazone obtains the ability of antiplatelet aggregation and maybe works in AA-induced selectively. Further study is worthy to be done for the many reasons.
